# Hydatid cyst of the breast: case report

**DOI:** 10.1016/j.ijscr.2020.10.109

**Published:** 2020-10-31

**Authors:** Kamal El Moussaoui, Amina Lakhdar, Aziz Baidada, Aicha Kherbach

**Affiliations:** Gynecology-Obstetrics and Endoscopy Department, Maternity Souissi, University Hospital Center IBN SINA, University Mohammed V, Rabat, Morocco

**Keywords:** Hydatid cyst, Breast cyst, Breast

## Abstract

**Introduction:**

The hydatid cyst of the breast is a rare pathology even in countries endemic to tuberculosis, however there remains a differential diagnosis evoked in front of any breast tumor. The clinic, mammography and ultrasound sometimes allow us to suspect the hydatid nature of the lesion. The definitive diagnosis remains histological and the treatment is always surgical. From one case, we recall the important role that cytology can play in preoparatory diagnosis.

**Case presentation:**

We report an observation of 59-year-old patient who lives in a tuberculosis endemic area of morocoo. the patient presented to our institute with palpable nodule in the right breast associated with pain of long duration. Clinical examination shows 2 masses occuping the entire upper outer quadrant of the right breast. Radiological explorations (mammography + ultrasound) objectified: A voluminous opacity of the QSE of the right breast corresponding on ultrasound to two cystic with anechoic content. the first classified ACR2 BIRADS. the second has ultimately echogenic content with a sloping portion producing the appearance of pseudo vegetation,classified ACR3 BIRADS. A fine needle aspiration of the cyst only showed the presence of altered polynuclear, without evidence of tumor cells. Treatment consisted of surgical excision such as perikystectomy and pathology examination demonstrated breast hydatidosis.postoperative evolution was favourable. Patient was put on medical treatment based on albendazole immediately.The patient was monitored for one year without local or distant recurrences.

**Conclusion:**

Although hydatid disease of the breast remains a rare localization of tuberculosis disease, it should nevertheless be considered as a differential diagnosis of breast tumors. Ultrasound, cytology and MRI play an important preoperative diagnostic role. Treatment is essentially based on surgical pericystectomy associated with medical treatment based on albendazole.

## Highlights

• Hydatid cyst should be considered as a differential diagnosis of all breast tumors.
• Ultrasound, cytology and MRI play an important preoperative diagnostic of hydatid cyst of the breast.
• Treatment of hydatid cyst of the breast essentially based on surgical pericystectomy associated with medical treatment based on albendazole.

## 1 Introduction

Hydatid cyst is a parasitic disease caused by the development in the body of the larval form of a tapeworm called echinococcus granulosus [1]. It was still considered a pulmonary pathology. however, nearly 17.9% of tuberculosis cases have extrapulmonary manifestations [2]. Breast and skin are considered rare sites of extrapulmonary mycobacterial infection, accounting for 0.1% to 0.5% of all. tuberculosis cases respectively [3]. Patients usually present to the hospital with a palpable, painless lump in the breast. Being difficult to differentiate it from other tumor lesions of the breast. Only a few cases are published in the literature and the majority of reported cases were diagnosed postoperatively [4]. From one case, we recall the epidemiological, diagnostic and therapeutic aspects of this pathology.

## 2 Case presentation

59-year-old patient, rural resident, who lives in a poor region of Morocco endemic to tuberculosis., menopausal for 5 years, without particular pathological history, was not taking any drugs and with no medical family history, consults for swelling in the right breast The onset of symptoms goes back 5 months by autopalpation of a nodule in the right breast associated with a progressive increase in its volume associated two months later with a mastodynia, without nipple discharge, without local inflammatory sign.

**Fig. 1:**
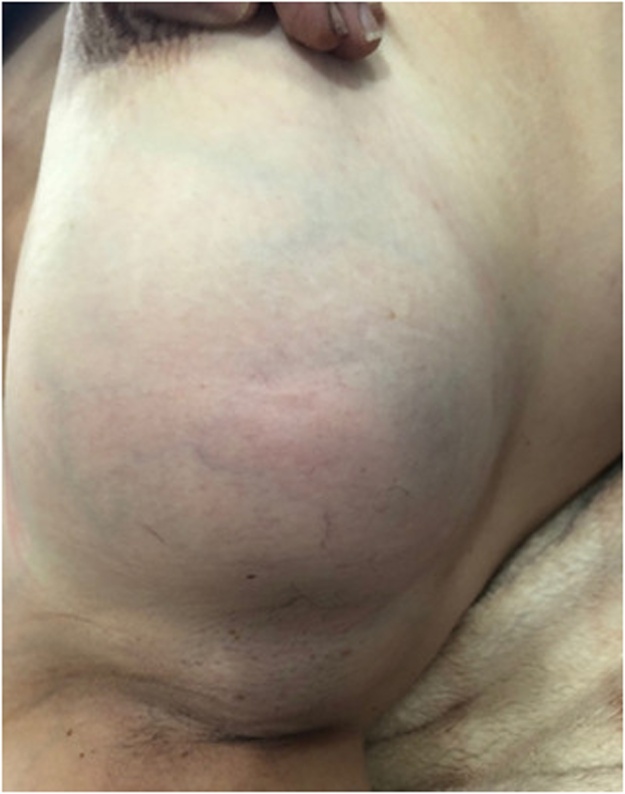
Picture showing two lumps in the right breast.

Clinical examination shows 2 masses occuping the entire upper outer quadrant of the right breast, respectively 8 cm, 4 cm in diameter and prolapsing in the axillary region, of soft consistency, well limited, mobile and painless, without inflammatory phenomenon of the skin integuments opposite. There is no axillary or supraclavicular lymphadenopathy, nor nipple discharge (Fig. 1).

**Fig. 2:**
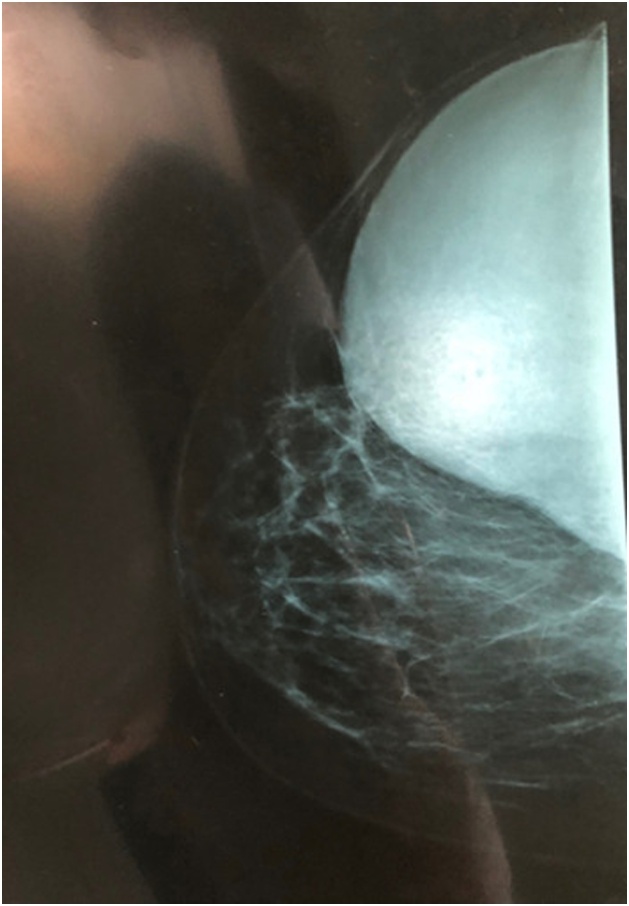
Craniocaudal views mammography of the right breast.

**Fig. 3:**
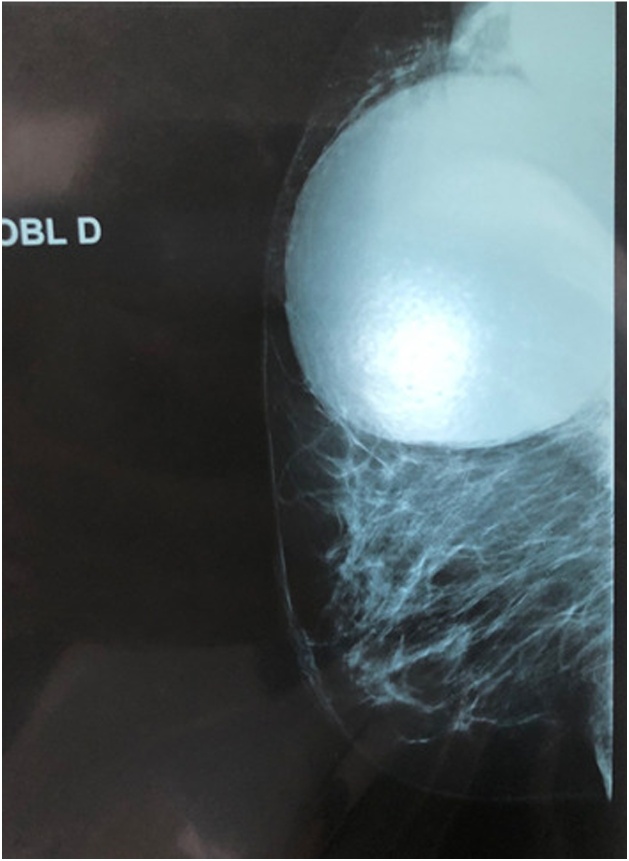
Mediolateral views mammography of the right breast.

Radiological explorations (mammography + ultrasound) (Figs. 2, 3) carried out objectified:

A voluminous opacity of the QSE of the dense and well limited right breast corresponding on ultrasound to two cystic masses of the QSE and the right axillary hollow.

- the first measures 66 × 84 mm in transverse diameter and has a thin wall and anechoic content. classified ACR2 BIRADS.
- The second measures 49 × 47 mm in transverse diameter in the axillary hollow and has ultimately echogenic content with a sloping portion producing the appearance of pseudo vegetation. classified ACR3 BIRADS.

The two masses are not vascularized by Doppler.

**Fig. 4:**
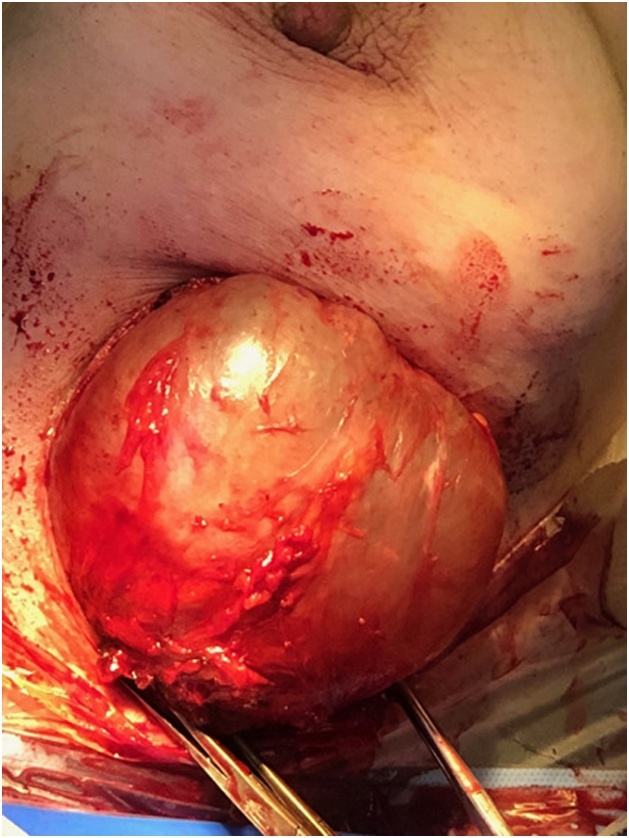
Intraoperative picture of the 2 hydatic cyst.

An abdominal ultrasound, a chest x-ray, as well as a biological blood test (complete blood count, ionogram) performed are completely normal. A fine needle aspiration of the cyst only showed the presence of altered polynuclear, without evidence of tumor cells. Treatment consisted of surgical excision such as perikystectomy (Fig. 4) who was performed by junior resident with 5 years of specialised training, and pathology examination demonstrated breast hydatidosis.

Postoperative evolution was favourable. Patient was put on medical treatment based on albendazole immediately. The patient was monitored for one year without local or distant recurrences.

## 3 Discussion

Hydatid disease is a cyclozoonosis caused by the larvae (metacestodes) in the stages of cestodes (tapeworms) belonging to the genus Echinococcus and the family Taenia. The disease exists in two forms: the larval stage (metacestode) and the adult stage (tapeworm). Parasites are perpetuated in life cycles with carnivores (dogs and wild canines) as definitive hosts. Humans are the accidental intermediate host (dead end) and animals (herbivores and omnivores) are both intermediate and definitive hosts [5]. The adult E. granulosus is a worm, when infected it produces eggs passed in the stool. Eggs ingested by intermediate hosts like cows, sheep, and humans release an embryo into the duodenum, which enters the intestinal lining and enters the portal circulation [6,7]. The liver acts as a first filter and shuts down about 75%, while the lungs, the second filter, shut down about 10% and only 15% of embryos are free to develop cysts in others. organs of the body.6 According to Barret and Thomas, 60% of cysts are found in the liver, 30% in the lungs,

2.5% in the kidneys, 2.5% in the heart and pericardium, 2% in the bones, 1.5% in the spleen, 1% in the muscle and 0.5% in the brain [8,9]. The embryo develops into a unilocular cyst [10].

Hydatid breast disease is extremely rare even in endemic areas; it may be the sole primary site or part of disseminated hydatidosis. Usually, the patient presents with a painless breast mass, which slowly increases in size without regional lymph node involvement. It usually affects women between the ages of 30 and 50. Clinically it could mimic a fibroadenoma, phyllodes tumors, chronic abscesses, or even carcinomas. So hydatid cyst of the breast should be included in the differential diagnosis of breast tumors especially in endemic areas [8,11,12].

Typically appears as a nodule of firm or persistent consistency, of variable size, with clear contours, mobile and often painless. It is sometimes calcified and without inflammatory phenomenon or lymphadenopathy. The cyst is infected poorly limited and pseudotumoral in 5% of cases thus simulating an abscess or possibly a malignant tumor [13,14].

Paraclinically, mammography and ultrasound may be helpful but not conclusive. Mammography may show a homogeneous, well-circumscribed lesion with ring-shaped structures within the mass [15]. When infection occurs secondarily, it is difficult to distinguish between hydatid cyst and breast abscess by mammography [16].

Ultrasound can visualize the cyst and define five types according to the Gharbi classification [17]. Types II and III hydatid cysts have more specific diagnostic imaging properties than other types. Multiple daughter cysts separated by a fluid matrix containing a mixture of broken daughter vesicle membranes, scolices and hydatid content of mixed echogenicity can give rise to a "wheel-spoke" appearance. Separation of the ruptured endocyst from the ectocyst layer leads to a floating membrane which gives the sign of the water lily [16].

However, less characteristic aspects can be diagnosed, raising suspicion of other pahologies, in particular tumors. In these cases, magnetic resonance imaging finds its place by showing an appearance of a well-circumscribed cystic lesion associated with capsular enhancement suggestive of hydatid cyst [18].

The extension workup consists of looking for other localizations, particularly hepatic and pulmonary, by chest x-ray and abdominal ultrasound [19].

Fine needle aspiration is performed whenever the cystic nature of the mass has been suspected [20,21]. It is preferably performed under ultrasound. In this case, it brings back a "rock water" liquid pathognomonic of mammary hydatidosis. In the case of a superinfected hydatid cyst, the fluid becomes purulent, and therefore the puncture takes on its full diagnostic value.

Biologically, hyper-eosinophilia is nonspecific. Casoni's intra-dermal reaction is only positive in 75% of cases. On the other hand, indirect immunofluorescence is a sensitive technique which gives good results [20–22].

The treatment of breast hydatidosis remains surgical. It consists of an excision of the cyst with pericystectomy which would protect against its break-in, a potential source of reinfestation. Some cases of postoperative recurrence have been reported but remain very rare. Only effective prophylaxis in order to break the cycle of E. granulosus will allow this pathology to regress [18].

This work has been reported in line with the SCARE 2018 criteria [23].

## 4 Conclusion

Although hydatid disease of the breast remains a rare localization of tuberculosis disease, it should nevertheless be considered as a differential diagnosis of breast tumors. Ultrasound, cytology and MRI play an important preoperative diagnostic role. Treatment is essentially based on surgical pericystectomy associated with medical treatment based on albendazole.

## Declaration of Competing Interest

The authors report no declarations of interest.

## Funding

There are no funding sources to be declared.

## Ethical approval

Ethics approval has been obtained to proceed with the current study. Consent to participate not applicable.

## Consent

Written informed consent was obtained from the patient for publication of this case report and accompanying images. A copy of the written consent is available for review by the Editor-in-Chief of this journal on request.

## Author contribution

KE made substantial contributions to conception and design, acquisition of data, analysis and interpretation of data; he has been involved in drafting the manuscript and revising it critically for important intellectual content. AL made substantial contributions to interpretation of data and she has beeninvolved in drafting the manuscript and revising it critically for important intellectual content. AB and AK made substantial contributions to conception and design and acquisition of data; they has been involved in drafting the manuscript. AK made substantial contributions to interpretation.

## Registration of research studies

NA.

## Guarantor

Corresponding author: Dr Kamal el Moussaoui.

The guarantor of this work, Oliver Scheufler, accepts full respon-sibility for the study and the conduct of the study, had access to thedata, and controlled the decision to publish.

## Provenance and peer review

Not commissioned, externally peer-reviewed.

## References

[1] Abi F., Kaiz D., Bouzidi A. (1989). Les localisations inhabituelles du kyste hydatique: à propos de 40 cas. J. Chir..

[2] Cowie R.L., Sharpe J.W. (1997). Extra-pulmonary tuberculosis: a high frequency in the absence of HIV infection. Int. J. Tuberculosis Lung Dis..

[3] Thompson K.S. (1997). Breast and cutaneous mycobacteriosis: diagnosis by fine-needle aspiration biopsy. Diagn. Cytopathol..

[4] Ali A., Aldhilan A., Makanjuola D., Abdulmohsen A. (2013). Preoperative diagnosis of hydatid cyst of the breast: a case report. Pan Afr. Med. J..

[5] Josef E.F., Miroslav Milicevic (2012). Echinococcal cyst-open approach.

[6] Garcia G., Shimizu R., Bruckner D. (1986). Sinus tract extension of liver hydatid cyst and recovery of diagnostic hooklets in sputum. Am. J. Clin. Pathol..

[7] Langer J.C., Rose D.B., Keystone J.S., Taylor B.R., Langer B. (1983). Diagnosis and management of hydatid disease of the liver. Ann. Surg..

[8] Das D.K., Choudhury U. (2002). An unusual breast lump. JIMA.

[9] Günag K., Müslümanoglu M., Taviloglu K. (1996). Hydatid cyst of the breast – avery rare form of hydatid disease. Breast Dis..

[10] Khatib I., Kharouf T., Khrais M., Khasawneh A. (2003). Primary hydatid cyst of the breast: report of two cases. Jordan Med. J..

[11] Farrokh D. (2000). Hydatid cysts of the breast: a report of three cases. Irn. J. Med. Sci..

[12] Acar T., Gömcel Y., Güzel K., Yazgan A., Aydyn R. (2003). Isolated hydatid cyst of the breast. SMJ.

[13] Essaidi H., Megdiche H., Rhimi Z., Missaoui M.N., Seghem M.H., Khari H. (1993). Kyste hydatique du sein: à propos de deux cas. Gynecologie.

[14] Jerbi M., Hidar S., Sahraoui W., Melloli R., Chaieb A., Bibi M. (2000). Kyste hydatique du sein: à propos d’un cas. Mag. Med..

[15] Vega A., Ortega E., Cavaoa A., Garijo F. (1994). Hydatid cyst of the breast: mammographic findings. Am. J. Roentgenol..

[16] Dagli A.F., Ozercan M.R., Kocakoc E. (2006). Hydatid cyst of the breast mimicking inflammatory carcinoma and mastitis. J. Ultrasound Med..

[17] Gharbi H.A., Hassine W., Brauner M.W., Dupuch K. (1981). Ultrasound examination of the hydatic liver. Radiology.

[18] Erkan N., Hazciyanli M., Yildirim M., Yilmaz C. (2004). A case report of unusual presence of hydatid disease in the pancreas and breast. JOP J. Pancreas.

[19] Delleur G., Tricoire J., Desaint G. (1980). Hydatidose mammaire: une observation. Nouv. Press. Med..

[20] Illoki L., Lefevbre G., Darbois Y., Tranbaloc P. (1992). Le kyste hydatique du sein : à propos d’un cas. Rev. Fr. Gynécol. Obstét..

[21] Ouedraogo E. (1986). Le kyste hydatique du sein : étude de 20 observations. J. Gynécol. Obstét. Biol. Rep..

[22] Benayed (1986). Kyste hydatique à localisation inhabituelle. Tunisie Méd..

[23] Agha R.A., Borrelli M.R., Farwana R., Koshy K., Fowler A., Orgill D.P., For the SCARE Group (2018). The SCARE 2018 Statement: Updating Consensus Surgical CAse REport (SCARE) Guidelines. Int. J. Surg..

